# Sub-lethal glyphosate exposure alters flowering phenology and causes transient male-sterility in *Brassica* spp

**DOI:** 10.1186/1471-2229-14-70

**Published:** 2014-03-21

**Authors:** Jason Paul Londo, John McKinney, Matthew Schwartz, Mike Bollman, Cynthia Sagers, Lidia Watrud

**Affiliations:** 1 USDA-ARS Grape Genetics Research Unit, Geneva, NY 14456, USA; 2 USEPA NHEERL Western Ecology Division, Corvallis, OR 97330, USA; 3 Department of Biological Sciences, University of Arkansas, Fayetteville, AR 72701, USA; 4 Department of Civil, Architectural, and Environmental Engineering, Missouri University of Science and Technology, Rolla, MO 65409, USA; 5 Oregon State University, Corvallis, OR 97330, USA

**Keywords:** Herbicide drift, Glyphosate, Glufosinate, Brassica, Transgene escape, Canola®

## Abstract

**Background:**

Herbicide resistance in weedy plant populations can develop through different mechanisms such as gene flow of herbicide resistance transgenes from crop species into compatible weedy species or by natural evolution of herbicide resistance or tolerance following selection pressure. Results from our previous studies suggest that sub-lethal levels of the herbicide glyphosate can alter the pattern of gene flow between glyphosate resistant Canola®, *Brassica napus*, and glyphosate sensitive varieties of *B. napus* and *B. rapa*. The objectives of this study were to examine the phenological and developmental changes that occur in *Brassica* crop and weed species following sub-lethal doses of the herbicides glyphosate and glufosinate. We examined several vegetative and reproductive traits of potted plants under greenhouse conditions, treated with sub-lethal herbicide sprays.

**Results:**

Our results indicate that exposure of *Brassica* spp. to a sub-lethal dose of glyphosate results in altering flowering phenology and reproductive function. Flowering of all sensitive species was significantly delayed and reproductive function, specifically male fertility, was suppressed. Higher dosage levels typically contributed to an increase in the magnitude of phenotypic changes.

**Conclusions:**

These results demonstrate that *Brassica* spp. plants that are exposed to sub-lethal doses of glyphosate could be subject to very different pollination patterns and an altered pattern of gene flow that would result from changes in the overlap of flowering phenology between species. Implications include the potential for increased glyphosate resistance evolution and spread in weedy communities exposed to sub-lethal glyphosate.

## Background

Agricultural land represents 11% of the total surface and 36% of the arable surface of the Earth [1] and continues to increase in an effort to feed a growing human population. As non-managed and marginal habitats are converted to agricultural use to meet this need, interactions between cultivated crops, associated anthropogenic selection pressures, and wild plant species increases. This interface represents a dynamic habitat where selection pressures may change quickly, creating a gradient of stress from lethal to survivable effects that contributes to adaptation and drives the evolution of tolerance and resistance traits. These forces may select for increased weediness traits in some plant species, impacting both wild and cultivated environments.

Herbicide drift is one of these selection pressures and occurs as a result of standard herbicide application practices near crop fields and management targets, but can also occur to a greater extent when proscribed herbicide application methods are not followed (e.g., application in high wind, unregulated weed control) [2]. As a result, sub-lethal concentrations of herbicides impact weedy or native plant communities at the crop-wild interface. The effect of any given dose of herbicide on a plant varies greatly with species. However, field and mesocosm tests of sub-lethal herbicide exposure demonstrate that herbicide drift can affect the plant community by reducing biomass and fecundity of both weedy and native plant species [3-5]. While herbicides are intended to kill weeds within crop fields, unintentional exposure at sub-lethal levels may result in the loss of species in wild and weedy habitats adjacent to crop fields, alter patterns of pollen movement between sexually compatible species, and change the relative contribution of different species to the seed bank [3,6-8]. Many factors contribute to the potential selective impact of sub-lethal herbicide exposure on weedy plant communities including: the genetic variation present within the community, plant community structure, developmental stage, inherent inter-specific tolerance differences, and acquired resistance via gene flow or selection [9]. Many different weedy species have been examined for their response to sub-lethal herbicide exposure and studies have shown that this selection pressure can be sufficient to drive the development of herbicide resistance. For example, exposure of weedy *Lolium* species to sub-lethal doses of ACCase herbicides has been shown to increase the level of resistance in progeny produced by surviving plants in as little as a single generation with dramatic gains in resistance in three generations both through inherited genes [10] and through acclimation mechanisms [11] such as delayed germination. While direct exposure to field application rates of herbicides would be expected to select for resistance conferred by genes of major effect, exposure to sub-lethal levels would be expected to select for polygenic resistance [9]. Weedy plant populations in field boundary habitats may be exposed to both strong and weak selection pressures, creating a scenario where resistance evolution might be optimized.

A study system where herbicide drift selection may occur outside of cultivated fields is the crop Canola® (*Brassica napus* L. [Brassicaceae]) and wild and weedy compatible species (see [12]) that overlap in distribution with Canola® cultivation. In the United States, Canola® production occurs primarily in the upper Midwest states of North Dakota, Minnesota, and Montana. Since their commercial release in Canada in 1995 and in the US in 1998, two types of transgenic Canola® have become dominant in Canola® agriculture and represent the vast majority of planted varieties [13]. Because of the overlap of compatible wild species with transgenic varieties, there is potential for transgene gene flow and hybridization between the crop and weedy species as well as selection for naturally evolved herbicide resistance in field boundary habitats.

The two types of transgenic Canola® most commonly cultivated are varieties resistant to the herbicides glufosinate-ammonium (Liberty Link®), and varieties resistant to glyphosate (Roundup Ready®). Glufosinate-ammonium is a contact herbicide that results in the inhibition of glutamine synthetase, resulting in disruptions to photosynthesis and leads to plant cell death [14,15]. In contrast, glyphosate is a systemic herbicide that upon contact with plant tissues is translocated within the plant to growing meristems. Glyphosate inhibits a key enzyme, EPSPS, in the shikimate pathway blocking the biosynthesis of several important amino acids and ultimately leads to plant death [16,17]. Because they each have a very different mode-of-action in target plants, these two herbicides are often applied in rotation in agricultural cropping systems. In fact, rotation of different herbicides is thought to delay the natural evolution of resistant weed populations by cycling selective pressures on in-field weed species [18].

We hypothesize that herbicide drift may affect the fitness and relative competitiveness of plants in a community by altering the flowering phenology of sensitive species without altering the phenology of resistant species. As a result, altered flowering phenology of sexually compatible feral crop and weed species may contribute to increased gene flow and hybridization between previously desynchronized plants, or decrease hybridization between previously synchronized plants [19]. In recent studies, we evaluated the effect of simulated drift of the herbicide glyphosate at a rate of 10% of field application levels in constructed plant communities composed of transgenic and non-transgenic *Brassica* species [19,20]. Observations of plants that were treated with glyphosate revealed that sensitive plants appeared to have a delay in development resulting in a change in flowering time. Presumably, a sub-lethal dose of glyphosate is sufficient to disrupt plant development without causing mortality. In addition, gene flow between certain *Brassica* spp. varieties in these experiments was significantly increased as a result of glyphosate drift [20]. Based on these observations, we conducted this study to test the hypothesis that sub-lethal doses of glufosinate and glyphosate change the flowering phenology and reproductive traits in *Brassica* spp.

## Methods

### Plant material and treatments

Seven different *Brassica* types (hereafter, varieties) were used in this study. These included three crop varieties of *Brassica napus*, two wild varieties of *Brassica rapa* L., and one wild variety of *Brassica nigra* L. and *Brassica juncea* L each. Two of the *B. napus* varieties were derived from a cv. Westar genetic background representing a single homozygous transgenic trait in glyphosate resistant Canola® (*B. napus* RR), and a non-transgenic segregating variety (*B. napus* null) [20]. The third *B. napus* variety used was the non-transgenic *B. napus* cv. Sponsor, which was included to determine if plant responses to herbicide drift can be generalized to Canola® cultivars with different genetic heritage. A transgenic glufosinate resistant variety of *B. napus* was not available for these studies. The remaining varieties included plants grown from seeds of two populations of *B. rapa* collected from weedy populations in Oregon and Northern California, a single population of *B. nigra* collected from a weedy population in Oregon, and a single population of *B. juncea* (PI649101), obtained from the USDA-GRIN national germplasm repository. The cultivated and wild species used here represent a portion of a hybridization complex between diploid (*B. rapa, B. nigra*) and tetraploid (*B. napus, B. juncea*) species [12]. *B. rapa* and *B. juncea* are sexually compatible with *B. napus* but represent self-incompatible and self-compatible modes of fertilization respectively. *B. nigra* has not been shown to be easily hybridized with *B. napus*[12] but shares a genome with the crop species. Additionally, *B. nigra* is frequently found as a weed in the production regions of the US (pers obs).

Plants were seeded in 15.24 cm (6 inches) diameter pots in standard potting media (Seedling Mix No. 1, OBC Northwest, Canby, OR) and cultivated in greenhouses at 20–30°C temperature and 16/8 hr day/night light regime. Two temporal replicate experiments were planted 2 weeks apart (June 10, 2009 and June 24, 2009) with variety groups randomized and rotated in position on separate greenhouse benches. Replicates were examined for a total of 100 days from the day of seeding encompassing the termination of flowering for the majority of plants under greenhouse conditions. Replicates were examined in the same greenhouse facility and plants were rotated in position on the greenhouse benches to assure environmental uniformity. Within each temporal replicate, 8 individually potted biological replicates of each variety were examined for each treatment except for *B. nigra* and *B. juncea* varieties, which suffered from variable germination. In replicate one, 6 biological reps per treatment/control were used for *B. juncea* while 4 reps per treatment and 6 reps for control were used for *B. nigra*. In replicate two, 7 replicates were used per treatment and 9 for control for *B. juncea,* while *B. nigra* had 8 replicates for all treatments/control. As a result, temporal replicate one had a total of 262 plants, while temporal replicate two had 277.

Four herbicide stress treatments were used. Treatments involved two brand-name herbicides, Liberty® (glufosinate-ammonium) and Roundup® (glyphosate, isopropylamine salt) applied at a simulated drift level concentration of 5% (0.05) and 10% (0.10) of the field application rate (f.a.r.) expected near Canola® agriculture: (glufosinate f.a.r. = 2.48 L/Ha; 0.05 = 0.12 L/Ha, 0.10 = 0.25 L/Ha; glyphosate f.a.r. = 2.34 L/Ha; 0.05 = 0.177 L/Ha, 0.01 = 0.234 L/Ha). Glufosinate treatments included ammonium sulfate in the spray mixture (3 lbs/acre) and glyphosate treatments included the surfactant “Preference” (0.5% v/v) following suggested rates. Treatments were applied using a track sprayer (Model RC5000-100EP, Mandel Scientific Company, Ltd. Guelph, Ontario, Canada). After herbicide applications had dried, plants were placed in the greenhouse and arranged in a randomized design to minimize spatial effects. Control plants were left unsprayed. Herbicide treatments were designed to simulate the drift of herbicides onto escaped crop and weed populations in adjacent non-crop habitats. As development times are variable between the varieties, herbicide drift treatments were applied 4 weeks after seeding. At this time, the majority of the varieties were either at the pre-bolting or bolting stage but no varieties had initiated flowering. No pollinators were released within the greenhouses, preventing unintentional cross-pollination of varieties. Non-transgenic, self-fertile varieties (*B. napus* and *B. juncea*) were not restricted in the development of seed pods (siliques).

### Data collection

Aboveground biomass (BIO), the total number of flowers (FA), the number of days to bolting (BOLT), days to first flower (DTF), and duration of flowering (DUR) were recorded for each individual plant. Days to first flower was recorded for all plants when the first flower-like structure with four petals was produced. Duration of flowering was recorded as the time from first flower to the termination of flowering (last fully formed flower) under greenhouse conditions. At the conclusion of flowering, plants were watered for 7 days before harvest to allow any developing siliques to elongate. At harvest, the number of flower attempts was counted by manually counting the siliques and pedicels on each raceme except for *B. nigra* due to the extremely large number of flowers on each plant of this species*.* Total aboveground biomass was collected and weighed after being dried in a 60°C drying oven (Blue M Model POM-326E, Thermal Product Solutions, New Columbia, PA) for 5 days.

Herbicide drift exposure could alter a plants ability to produce seeds either by impacting male function, female function, or both. For self-fertile species (*B. napus, B. juncea*), we evaluated the impact of herbicide treatments on reproduction by measuring the proportion of successful siliques vs. unsuccessful siliques. Measurements of successful self-fertility cannot distinguish reductions in reproductive fitness that arise either due to impacts on the stamen or on the pistil. Additionally, *B. rapa* and *B. nigra* varieties in this experiment are self-incompatible so additional measures of male and female function were conducted. Herbicide effects on male function were evaluated by digital photography and image analysis of anther morphology. Anthers were collected from the stamens of all varieties in all treatments from at least three flowers per plant, and three plants per treatment. Twenty-one days after herbicide applications, anthers were sampled from freshly opened flowers and placed in a 5% sucrose solution and MTT viability stain [21]. We attempted to assess pollen viability with the viability stain, however, complications with pollen extraction from the deformed anthers obtained from glyphosate treated plants precluded quantitative measures of pollen viability. Instead, we quantified morphological deformities by measuring the anther length (L), width (W), and the W/L ratio (R) from prepared slides. Image analysis was conducted using ImageJ Software [22].

To evaluate female function, manual pollinations were performed between *B. napus* cv. RR as a paternal parent and *B. napus* cv. Null, *B. napus* cv. Sponsor, and *B. rapa* OR as maternal parents. Crosses were not performed on *B. rapa* CA or *B. juncea* due to low sample sizes of recovered flowers, nor were crosses made to *B. nigra* due to high incompatibility with *B. napus*[12]. Pistils were hand pollinated at 10 days post treatment to assess the viability of pistils on plants in the early stages of recovery from herbicide drift. At 21 days post treatment, a second evaluation of pistil function on the same plants was conducted. The second evaluation corresponded to the time at which “recovered” flowers were observed. At least 3 individual flowers were pollinated on at least three plants in each treatment. Due to limited available pistils on *B. napus* plants at both pollination time points, it was necessary to pool the manual pollinations for cv. Null and cv. Sponsor varieties. The percent of successful manual pollinations was used to determine the viability of pistils at both the pre-recovery (10 day) and post-recovery (21 day) time points.

Data was initially analyzed as multivariate data with MANOVA but due to a lack of correlation between response variables (data not shown), data were further analyzed with ANOVA (PROC GLM) using SAS 9.2 (SAS/STAT). The two different herbicide types were examined using contrast statements for comparisons to control. Our experimental factors included Treatment (T), Variety (V), and Rep (R); all interaction effects were tested and included TxV, TxR, RxT, and TxVxR. When interactions were significant, examination of the simple treatment effects was performed [23]. Pistil viability measurements were analyzed using a nonparametric Mann–Whitney Wilcoxon Test in R [24].

## Results

Significant interactions between main effects were observed (Additional file 1: Table S1) indicating varieties should be examined separately. A significant glyphosate x variety interaction was expected due to inclusion of the glyphosate resistant *B. napus* cv. RR. The second temporal replicate had significantly longer average days to flower, shorter duration of flowering, reduced number of flowers per plant and lower biomass than temporal replicate one for most varieties (data not shown). However, the differences between temporal replicates did not result in differences in the response of varieties to herbicide treatments but instead the magnitude of the effect of glyphosate treatment was greater in the second replicate (data not shown). Measurements from the two replicates were thus combined for analyses of treatment effects and varieties were examined for effects of treatment in contrast to control values (Table 1).

**Table 1:** ANOVA results for plant measurements in response to glyphosate treatments separated for effects of 0.05 and 0.1 levels of glyphosate

	**Structure**				**Phenology**						**Male reproduction**						**Pistil function**				**Self fertility**	
	**BIO**		**FA**		**BOLT**		**DTF**		**DUR**		**Anther L**		**Anther W**		**Anther R**		**10d**		**21d**		**Siliques**	
**Variety**	**0.05**	**0.1**	**0.05**	**0.1**	**0.05**	**0.1**	**0.05**	**0.1**	**0.05**	**0.1**	**0.05**	**0.1**	**0.05**	**0.1**	**0.05**	**0.1**	**0.05**	**0.1**	**0.05**	**0.1**	**0.05**	**0.1**
*B. napus cv. RR*	-	-	-	-	-	-	-	-	-	-	-	-	-	**0.031**	**0.041**	**0.040**	na	na	na	na	na	na
*B. napus cv. Null*	-	-	**0.030**	-	-	-	**<0.001**	**<0.001**	-	-	**0.001**	**<0.001**	-	-	**<0.001**	**<0.001**	-	**0.026**	-	**0.006**	**<0.001**	**<0.001**
*B. napus cv. Sponsor*	-	-	**0.003**	-	-	-	**<0.001**	**<0.001**	-	-	**<0.001**	**<0.001**	-	-	**<0.001**	**<0.001**	na	na	na	na	**<0.001**	**<0.001**
*B. rapa OR*	-	-	-	-	-	-	**<0.001**	**<0.001**	**0.001**	**0.001**	**0.001**	**<0.001**	-	**<0.001**	**0.005**	**0.013**	**0.025**	**0.001**	**0.006**	**<0.001**	na	na
*B. rapa CA*	-	-	-	-	**0.028**	-	**0.009**	**0.001**	-	-	-	**0.019**	-	-	-	**0.031**	na	na	na	na	na	na
*B. juncea*	-	-	-	-	**<0.001**	-	**<0.001**	**<0.001**	-	-	**<0.001**	**<0.001**	**0.008**	**0.005**	-	**0.002**	na	na	na	na	**<0.001**	**<0.001**
*B. nigra*	-	**0.008**	na	na	-	**0.022**	**0.001**	**<0.001**	-	**0.005**	-	**0.001**	-	-	**0.003**	**0.015**	na	na	na	na	na	na

Response variables: vegetative biomass (BIO) flower attempts (FA), changes in bolting (BOLT), days to flower (DTF), duration of flowering (DUR), male reproductive measures, pistil function, and self-fertility. Values in boldface type indicate significance at P < 0.05. Lack of a value indicates no significance and na indicates no measurement taken.

### Glufosinate treatments

**Figure 1:**
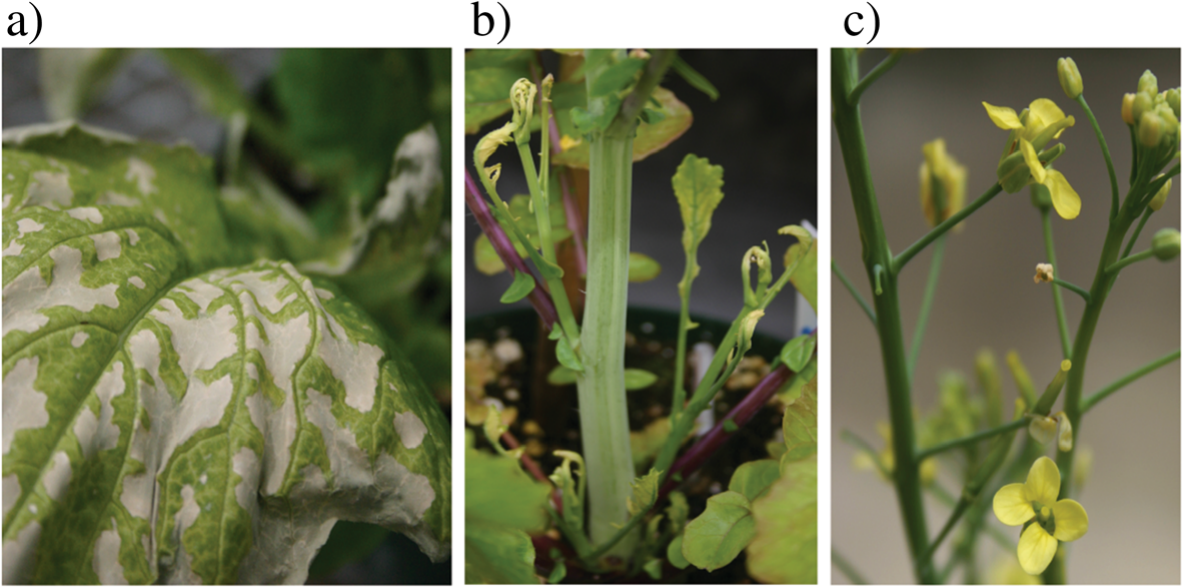
**Effects of herbicide drift damage on *Brassica*. a)** Necrotic lesions at site of contact due to glufosinate application. **b)** Misshapen and stunted meristems due to systemic toxicity at growing tissues following glyphosate application. **c)** Malformed and male-sterile “recovered” flowers that develop after plant recovery from glyphosate applications. Note the lack of anthers.

Plants that were exposed to glufosinate developed contact damage on vegetative tissues, observed as chlorotic and necrotic lesions, within the first few days after treatment (Figure 1a). After the initial plant damage, glufosinate treated plants resumed vegetative and reproductive growth without any further morphological indication of toxicity.

**Figure 2:**
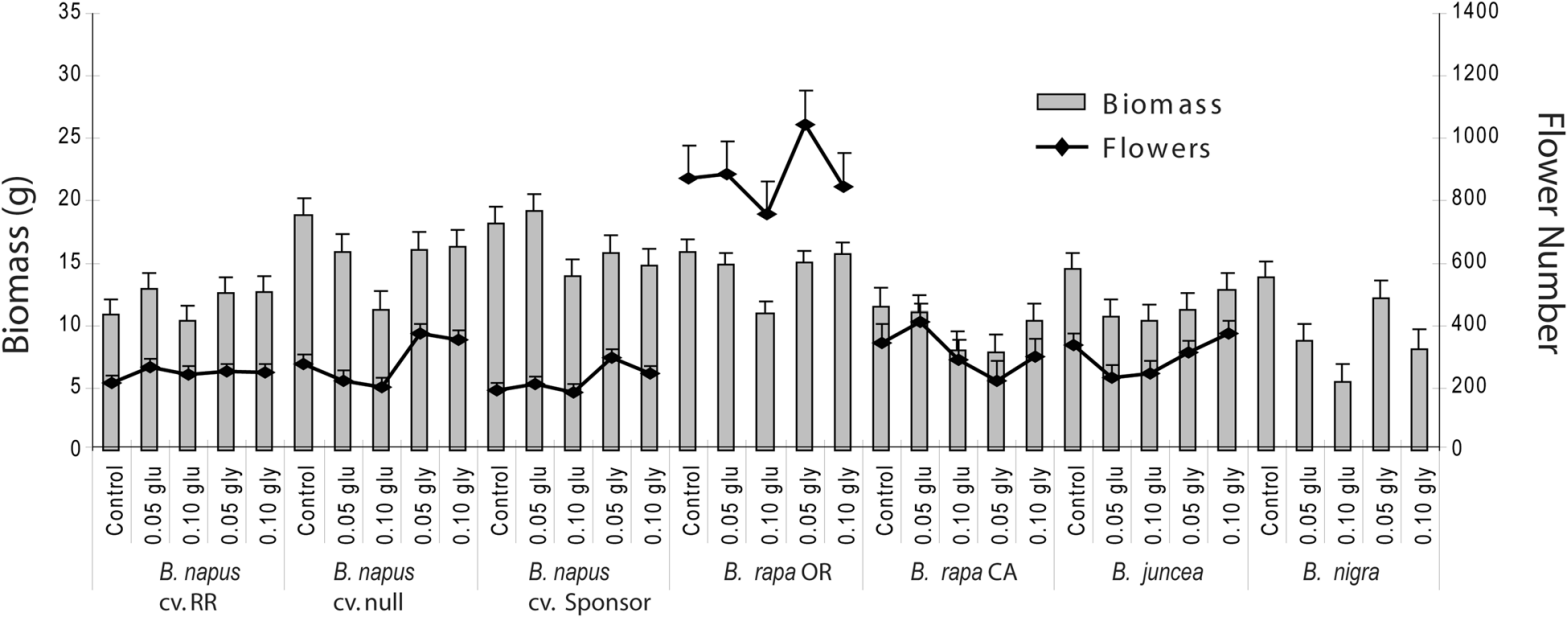
**Effect of herbicide applications on plant biomass (BIO) and flowering attempts (FA).** Left hand axis represents plant biomass, right axis represents the number of flowers produced. Bars and points represent mean values combined from both replicates. Treatments denoted on x axis; glu = glufosinate, gly = glyphosate. Error bars represent +/− one SE.

Glufosinate treatment effects were primarily limited to the plant structure responses of aboveground biomass and a single effect on flower attempts. Glufosinate treatments significantly reduced the biomass produced by *B. napus* cv. Null (0.1; p = 0.004)*, B. rapa* OR (0.1; p = 0.0005), *B. juncea* (0.05; p = 0.04, 0.1; p = 0.02), and *B. nigra* (0.05; p = 0.0087, 0.1; p < 0.001) with the greatest reduction in biomass at the 0.10 drift level. The remaining three variety biomass measures were not significantly reduced though the data trended toward reductions at the 0.10 level (Figure 2). Glufosinate treatments did not have a consistent effect on any other plant response (data not shown).

### Glyphosate treatments

Plants exposed to glyphosate demonstrated evidence of herbicide damage as stunting, deformation, and chlorosis of meristems after treatment (Figure 1b). The development of inflorescence meristems was halted in all sensitive varieties. After a variety-specific time delay, the primary meristem and additional secondary meristems resumed development. Flowers that formed following treatment exposure were observed as deformed flower-like structures with shrunken, pale petals; these structures typically lacked stamens (Figure 1c). Pistil morphology appeared to be more resistant to glyphosate damage, and normal pistils were nearly always present on post-treatment flowers.

In contrast to glufosinate, glyphosate treatments produced significant changes in all plant responses measured. Glyphosate treatments reduced the biomass of the weedy *B. nigra* species at the 0.10 concentration. Glyphosate treatments also resulted in significantly greater flower attempts on both sensitive *B. napus* cultivars and at both 0.05 and 0.10 treatment levels (Table 1, Figure 2), possibly indicating a stimulatory effect of low levels of glyphosate on flower production in *B. napus*. Increased flower numbers were not observed for other varieties.

**Figure 3:**
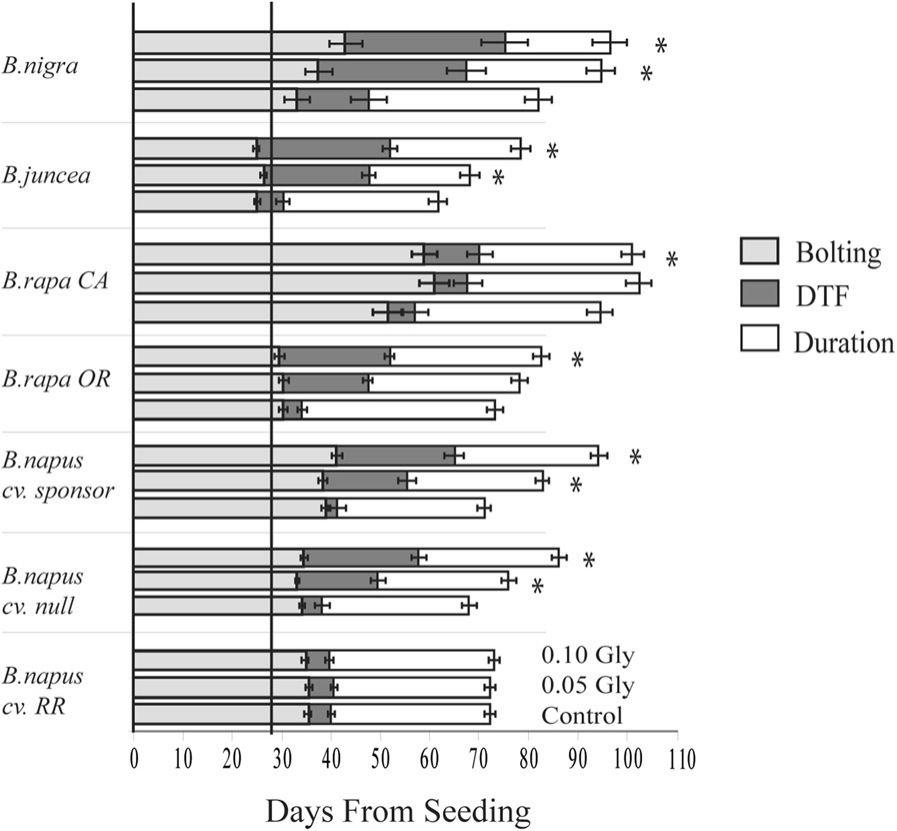
**Changes in Bolting, Days to Flower (DTF), and Duration of flowering on *Brassica *resulting from glyphosate applications.** Treatments indicated for each variety, ordered as labeled for *B. napus* cv. RR Error bars indicate +/− one standard error. Asterisk indicates significant change in DTF at P < 0.05. Vertical line at 28 days indicates time of glyphosate application.

Effects of glyphosate were assessed on the flowering phenology pattern of *Brassica* spp. by examining the days to bolting (BOLT), days to first flower (DTF), and the duration of flowering (DUR) (Figure 3). Glyphosate treatments significantly impacted all three of these measurements though not for every variety. Glyphosate treatments significantly delayed the days to bolting for *B. rapa* CA, *B. juncea* and *B. nigra* varieties (Table 1, Figure 3). Glyphosate treatments significantly delayed flowering in all of the varieties except for the glyphosate resistant transgenic *B. napus* cv. RR (Table 1). Flowering delays were different for each of the varieties with *B. rapa* CA having the shortest delay, 10.70 days at 0.05 glyphosate, and *B. nigra* having the longest delay, 29.46 days at 0.10 glyphosate. The delayed recovery in flowering was more pronounced at the higher drift concentration (0.10) for all six sensitive varieties (Figure 3, Additional file 2: Table S2). Glyphosate treatments also significantly reduced the duration of flowering for *B. rapa* OR, and *B. nigra*.

**Figure 4:**
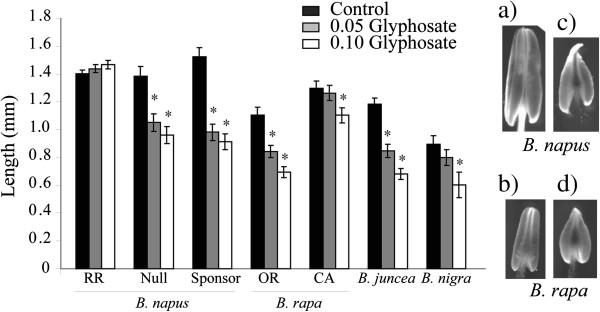
**Effect of glyphosate treatments on anther length.** Significant decreases in length denoted by asterisk at P < 0.05. Digital photo of anthers from an untreated **a) ***B. napus* and **b) ***B. rapa* and anthers **c)**, **d)** from 0.10 glyphosate treatments.

Male and female reproductive attributes were examined separately to determine if glyphosate drift toxicity affects male and female function differently. Glyphosate treatments typically resulted in deformed and shortened anthers that appear to be unable to properly dehisce and release pollen (Figure 4). Anther length was significantly reduced in all varieties except the glyphosate resistant *B. napus* cv. RR variety. Anther width was less sensitive to glyphosate effects and significantly increased for *B. rapa* OR, *B. juncea*, and *B. napus* cv. RR varieties. Consequently, the anther ratio was significantly different in all varieties (Table 1) at the 0.10 treatment level.

**Figure 5:**
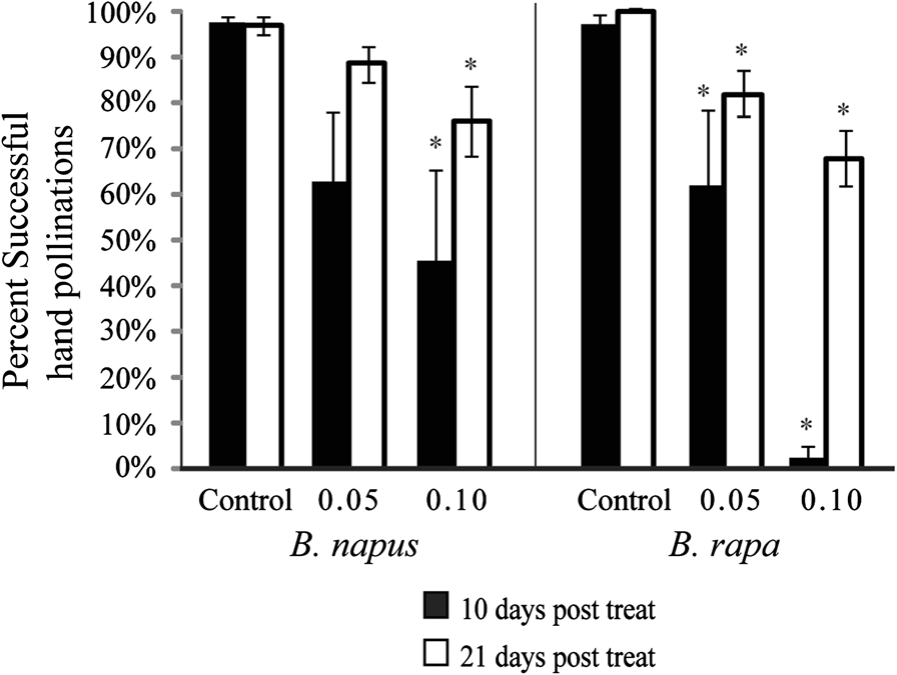
**Effect of glyphosate treatments on pistil function, evaluated by successful hand pollinations.** Significant differences noted with an asterisk. Error bars indicate +/− one SE.

Pistil function was sensitive to glyphosate drift treatments. Pre-recovery pistils had significantly reduced function for *B. napus* at the 0.10 treatment level and both 0.05 and 0.01 treatment levels for *B. rapa* (Figure 5). Pistils that were pollinated after plants appeared to have resumed normal flowering had much higher function in both *B. napus* and *B. rapa*, though function remained lower than pollinations made on control plants (Figure 5).

**Figure 6:**
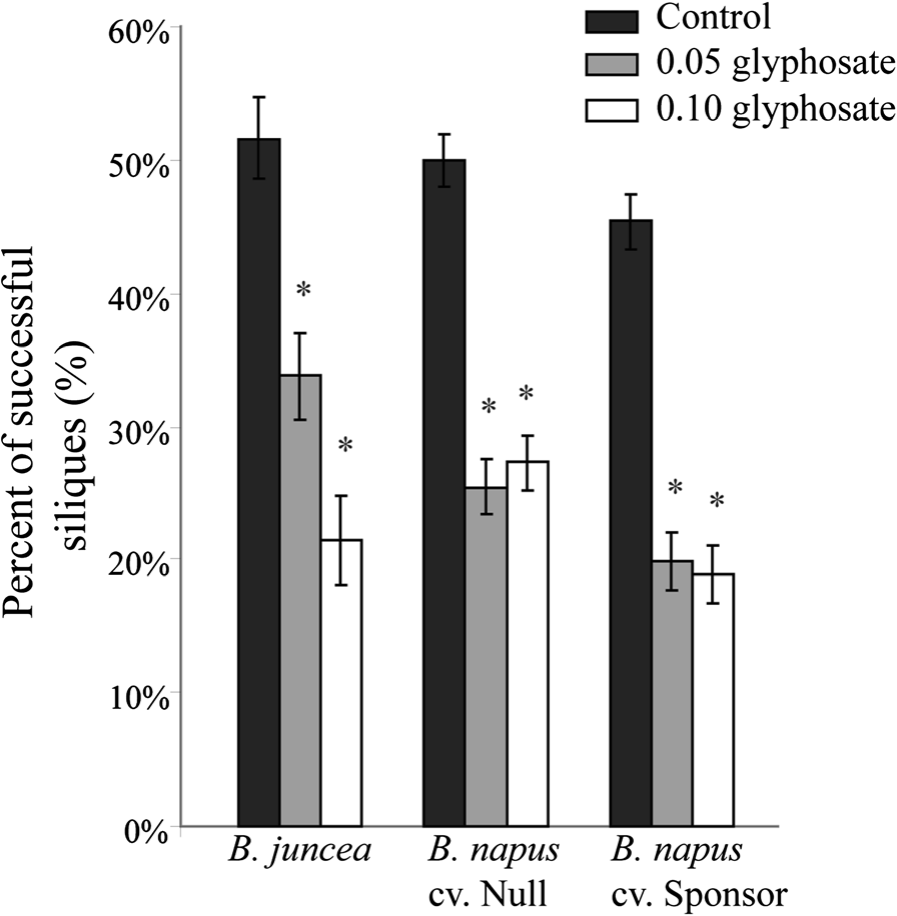
**Assessment of self-fertility changes due to glyphosate treatments.** Percent of successful siliques produced on self-fertile varieties. Asterisk indicates P < 0.05. Error bars represent +/− one SE.

The ability of plants to self-fertilize was examined on the two sensitive *B. napus* cultivars and the *B. juncea* variety. All three varieties were similar under control conditions, producing approximately 49% ± 1% of flowers as siliques. The proportion of flowers that successfully formed a silique was significantly lower with glyphosate treatment, with a reduction of approximately 50% for all three varieties in both 0.05 and 0.10 treatment levels (Figure 6).

## Discussion

Exposure of *Brassica* species to sub-lethal herbicide results in changes in biomass, flowering phenology, and reproductive function. Of the two herbicides tested here, only glyphosate exposure resulted in changes in flowering time and reproductive function. Plants that were exposed to sub-lethal glyphosate demonstrated variable delay in flowering time and all delays were significantly greater than unsprayed or glyphosate resistant plants. Male reproductive function was much more sensitive to glyphosate exposure than female function and as a result, plants were rendered functionally out-crossing for a significant period of time (2–4 weeks). These results demonstrate the potential for sub-lethal glyphosate to alter flowering time in glyphosate sensitive plant populations. These changes in phenology in wild and weedy plants could contribute to changes in gene flow patterns between resistant and sensitive plants such that resistance alleles may unidirectionally move from resistant plants into functionally out-crossing sensitive plants. Changes in the flowering phenology and reproductive strategy of plants, specifically in feral conventional crops or sexually compatible weeds, could have important implications for transgene confinement and management.

The results of our study demonstrate the differential effects of sub-lethal herbicide exposure and highlight the potential for ecological and evolutionary impacts in weedy plant communities. Evolution of herbicide resistance in weedy plant species is perhaps the greatest concern in regards to weed management [9]. Currently there are 221 different species that are considered herbicide resistant and weeds have evolved resistance to 152 different herbicides [25]. Evidence for evolution of resistance has been observed for both major gene traits as well as multigenic traits. Direct exposure to herbicide spray, such as would occur within crop fields, is expected to favor evolution of major effect genes that rise to fixation in the weed population quickly [26]. In contrast, low and sub-lethal exposure would act to favor resistance traits that include many different loci that could combine to increase resistance following outcrossing between surviving plants [10,27]. For example, the evolution of glyphosate resistant *Lolium* spp. in vineyards and orchards [28] following repeated, non-lethal exposures. In this study, we observed a potential interaction between sub-lethal exposure and herbicide resistance in weedy plant species. The implications of this interaction are that direct exposure to glyphosate would favor feral resistant crop plants in weedy communities, suppressing growth and survival of sensitive varieties while increasing the representation of resistant seeds in the weed seed bank. Sub-lethal exposure may also enhance the movement of transgenic resistance traits between plants through the synchronization and de-synchronization of flowering periods between resistant and sensitive species, creating a window of optimal out-crossing. Selective sterility of male tissues, but partially/fully functional female tissues in sensitive species would explain the results of our previous study [20] where outcrossing rate was seen to significantly increase for non-transgenic Canola® varieties exposed to sub-lethal glyphosate treatments. Expanded upon further, this temporary enhancement of pollen based gene flow between resistant crop varieties and sensitive weed varieties might result in the increased production of hybrid seed on receptive weed plants, impacting the structure and identity of the future weed seed bank. These hybrid seeds may then germinate and have a selective advantage in subsequent generations exposed to herbicide drift, contributing to the preservation of resistance alleles in the weed population.

Several other studies have uncovered results similar to ours in regard to the transient and specific loss of function of the male reproductive structures suggesting that sub-lethal glyphosate effects are not unique to *Brassica* species. Studies in morning glory have revealed population variability in the survival of glyphosate application and surviving plants often have functionally female flowers due to abnormal stamens [29]. Similarly, studies in cotton have shown glyphosate-induced changes in microtubules in anthers, leading to poor dehiscence [30], possibly similar to the mechanism contributing to the reduction in anther dehiscence we observed. Glyphosate resistant corn and cotton varieties that have reduced transgene expression in male tissues also suffer from shortened anther filaments leading to reduced pollen transfer between anther and pistil [31] and reduced pollen viability [32]. Interestingly, early studies of glyphosate’s mode of action demonstrated the function of glyphosate sprays used as a male specific gametocide for preventing the self-pollination of wheat cultivars [33]. It appears that while the utility of glyphosate application for male gametocidal action is well known, the implications of this effect regarding gene flow in the environment between compatible species remain understudied. Though additional studies are needed, it is likely that sub-lethal glyphosate exposure has the potential to alter the flowering phenology and mating system function of many different wild and weedy species.

Future studies are necessary to evaluate and describe the level of herbicide drift occurring in weedy plant populations. While data exists on rates of herbicide drift under prescribed best practices [2], less data are available that describes the rates of non-regulated herbicide exposure and applications under adverse conditions (e.g., windy conditions). Additionally, little attention is paid by the majority of weed evolution studies on weeds that grow just beyond agricultural fields. Instead, it is assumed that direct exposure to herbicides is the dominant selection pressure contributing to herbicide resistance evolution. Field studies including multi-species plots, exposed to varied herbicide levels over different developmental stages would further add refinement to the potential implications of sub-lethal herbicide exposure.

## Conclusions

In conclusion, we argue that sub-lethal herbicide exposure outside of fields may contribute to the rise of resistant weeds and our study demonstrates the potential mechanism for such resistance evolution. Our results demonstrate that sub-lethal exposure to these two herbicides results in different potential for population level impacts. Namely, populations exposed to sub-lethal glyphosate may experience changes in flowering phenology that may lead to altered rates of inter and intra-specific gene flow. As a result of repeated exposure, it is possible that resistance could evolve via selection on standing variation in weed populations or through direct transfer of transgenic resistance traits due to alterations in flowering phenology and transient male-sterility.

## Competing interests

The authors declare that they have no competing interests.

## Authors’ contributions

JL, MB, JM, and MS carried out the greenhouse phenotypic measurements and manual crosses to evaluate reproductive function. JL conceived and designed the study and performed the statistical analysis. MB, CS, and LW assisted in the design and coordination of the study and helped to draft the manuscript. All authors have read and approved the final manuscript.

## Supplementary Material

Additional file 1: Table S1 MANOVA results for plant response variables: changes in bolting (BOLT), days to flower (DTF), duration of flowering (DUR), vegetative biomass (BIO), flower attempts (FA), anther length (L), anther width (W), anther ratio (R) and self fertility (SF). Values in boldface type indicate significance at P < 0.05. Reduced degrees of freedom for FA and Self Fertility values are due to reduced varieties for these measurements. No measures of FA were taken for *B. nigra* and measures of SF were only taken for null and Sponsor varieties of *B. napus*. Click here for file

Additional file 2: Table S2 Days to first flower (DTF) following glyphosate applications. Change in days is relative to untreated (control) plants. +/- indicates one standard error (SE). Click here for file
