# Supplementary material for: Sub-lethal glyphosate exposure alters flowering phenology and causes transient male-sterility in Brassica spp
Source: BMC Plant Biol. 2014 Mar 21;14:70. doi: 10.1186/1471-2229-14-70 (PMC3998022; doi:10.1186/1471-2229-14-70)
Supplement: Additional file 1: Table S1 — MANOVA results for plant response variables: changes in bolting (BOLT), days to flower (DTF), duration of flowering (DUR), vegetative biomass (BIO), flower attempts (FA), anther length (L), anther width (W), anther ratio (R) and self fertility (SF). Values in boldface type indicate significance at P < 0.05. Reduced degrees of freedom for FA and Self Fertility values are due to reduced varieties for these measurements. No measures of FA were taken for B. nigra and measures of SF were only taken for null and Sponsor varieties of B. napus. [file 1471-2229-14-70-S1.docx]

**Supplemental Table 1.** MANOVA results for plant response variables: changes in bolting (BOLT), days to flower (DTF), duration of flowering (DUR), vegetative biomass (BIO), flower attempts (FA), anther length (L), anther width (W), anther ratio (R) and self fertility (SF). Values in boldface type indicate significance at P<0.05. Reduced degrees of freedom for FA and Self Fertility values are due to reduced varieties for these measurements. No measures of FA were taken for *B. nigra* and measures of SF were only taken for null and Sponsor varieties of *B. napus*.

|  |  | Phenology | | | | | | Structure | | | | | Reproduction | | | | | | | | | |
| --- | --- | --- | --- | --- | --- | --- | --- | --- | --- | --- | --- | --- | --- | --- | --- | --- | --- | --- | --- | --- | --- | --- |
|  |  | BOLT | | DTF | | DUR | | BIO | |  | FA | | Anther L | | Anther W | | Anther R | |  | Self-Fertility | | |
|  | df | F | p | F | p | F | p | F | p | df | F | p | F | p | F | p | F | p | df | F | p | |
| Glufosinate (T) | 2 | 0.84 | 0.432 | 0.6 | 0.551 | 0.12 | 0.885 | **22.5** | **<0.001** | 2 | 2.18 | 0.116 | 1.69 | 0.19 | 0.68 | 0.510 | 0.47 | 0.624 | 2 | 0.86 | 0.355 | |
| Variety (V) | 6 | 125.02 | **<0.001** | 101.93 | **<0.001** | 22.92 | **<0.001** | **16.04** | **<0.001** | 5 | **77.44** | **<0.001** | 127.33 | **<0.001** | 52.74 | **<0.001** | 14.2 | **<0.001** | 2 | 7.28 | **0.008** | |
| V*T | 12 | 1.25 | 0.246 | 1.85 | **0.041** | 1.42 | 0.159 | **1.91** | **0.034** | 10 | 0.72 | 0.709 | 1.34 | 0.209 | 1.84 | 0.053 | 1.35 | 0.204 | 4 | .428 | 0.513 | |
| Rep (R) | 1 | 1.01 | 0.316 | 7.52 | **0.007** | 10.78 | **0.001** | **6.04** | **0.015** | 1 | **10.07** | **0.002** | 17.98 | **<0.001** | 3.81 | 0.054 | 4.14 | **0.045** | 1 | 3.08 | 0.081 | |
| R*T | 2 | 2.63 | 0.074 | 0.45 | 0.640 | 0.68 | 0.510 | 0.1 | 0.906 | 2 | 1.49 | 0.227 | 4.69 | **0.012** | 5.65 | **0.005** | 4.05 | **0.021** | 2 | 0.07 | 0.791 | |
| R*V | 6 | 0.91 | 0.485 | 5.56 | **<0.001** | 2.03 | 0.062 | **6.29** | **<0.001** | 5 | **4.4** | **0.001** | 1.28 | 0.274 | 5.32 | **<0.001** | 4.32 | **0.001** | 2 | 4.69 | **0.032** | |
| R*V*T | 12 | 1.62 | 0.087 | 0.96 | 0.491 | 0.99 | 0.459 | 0.95 | 0.493 | 10 | 1.67 | 0.089 | 1.33 | 0.216 | 4.04 | **<0.001** | 1.86 | 0.050 | 4 | 0.16 | 0.693 | |
|  |  |  |  |  |  |  |  |  |  |  |  |  |  |  |  |  |  |  |  |  |  | |
|  |  |  |  |  |  |  |  |  |  |  |  |  |  |  |  |  |  |  |  |  |  | |
|  |  | Phenology | | | | | | Structure | | | | | Reproduction | | | | | | | | | |
|  |  | BOLT | | DTF | | DUR | | BIO | |  | FA | | Anther L | | Anther W | | Anther R | |  | Self-Fertility | | |
|  | df | F | p | F | p | F | p | F | p | df | F | p | F | p | F | p | F | p | df | F | | p |
| Glyphosate (T) | 2 | 4.26 | **0.015** | 208.17 | **<0.001** | 24.03 | **<0.001** | 3.72 | 0.026 | 2 | 1.14 | 0.322 | 66.53 | **<0.001** | 4.62 | **0.012** | 35.47 | **<0.001** | 2 | 130.22 | | **<0.001** |
| Variety (V) | 6 | 130.49 | **<0.001** | 88.76 | **<0.001** | 11.84 | **<0.001** | 11.88 | **<.0001** | 5 | 83.51 | **<0.001** | 49.86 | **<0.001** | 25.63 | **<0.001** | 6.05 | **<0.001** | 2 | 9.24 | | **0.000** |
| V*T | 12 | 2.25 | **0.010** | 10.12 | **<0.001** | 4.3 | **<0.001** | 1.22 | 0.269 | 10 | 1.28 | 0.240 | 5.43 | **<0.001** | 2.56 | **0.005** | 3.1 | **0.001** | 4 | 2.69 | | **0.034** |
| Rep (R) | 1 | 14.68 | **<0.001** | 80.18 | **<0.001** | 15.14 | **<0.001** | 13.43 | **<0.001** | 1 | 8.96 | **0.003** | 3.06 | 0.083 | 0.18 | 0.670 | 1.84 | 0.177 | 1 | 22.76 | | **<0.001** |
| R*T | 2 | 0.41 | 0.666 | 15.32 | **<0.001** | 0.97 | 0.379 | 1.2 | 0.302 | 2 | 4.22 | **0.016** | 3.45 | **0.036** | 0.76 | 0.470 | 3.47 | **0.035** | 2 | 10.3 | | **<0.001** |
| R*V | 6 | 6.97 | **<0.001** | 11.99 | **<0.001** | 5.1 | **<0.001** | 4.48 | **<0.001** | 5 | 6.95 | **<0.001** | 4.44 | **0.001** | 3.25 | **0.006** | 1.37 | 0.236 | 2 | 7.46 | | **0.001** |
| R*V*T | 12 | 0.89 | 0.562 | 2.55 | **0.003** | 1.65 | 0.079 | 0.81 | 0.638 | 10 | 1.43 | 0.166 | 0.93 | 0.517 | 0.92 | 0.535 | 1.33 | 0.212 | 4 | 3.85 | | **0.006** |
