# Supplementary material for: Sub-lethal glyphosate exposure alters flowering phenology and causes transient male-sterility in Brassica spp
Source: BMC Plant Biol. 2014 Mar 21;14:70. doi: 10.1186/1471-2229-14-70 (PMC3998022; doi:10.1186/1471-2229-14-70)
Supplement: Additional file 2: Table S2 — Days to first flower (DTF) following glyphosate applications. Change in days is relative to untreated (control) plants. +/- indicates one standard error (SE). [file 1471-2229-14-70-S2.docx]

**Supplemental Table 2)** Days to first flower (DTF) following glyphosate applications. Change in days is relative to untreated (control) plants. +/- indicates one standard error (SE).

| Flowering Delay (Days) | | Control DTF | | Glyphosate DTF | | | | | |
| --- | --- | --- | --- | --- | --- | --- | --- | --- | --- |
|  |  |  |  | 0.05x | | Difference | 0.1x | | Difference |
| *B. napus* cv. RR | | 40.13 | ± 0.45 | 40.69 | ± 0.77 | 0.56 | 40.13 | ± 0.43 | 0.01 |
| *B. napus* cv. Null | | 38.38 | ± 0.43 | 49.56 | ± 1.80 | 11.19 | 57.81 | ± 2.56 | 19.44 |
| *B. napus* cv. Sponsor | | 40.31 | ± 0.65 | 54.53 | ± 1.97 | 14.22 | 65.19 | ± 3.16 | 24.88 |
|  | *Average B. napus* |  |  |  |  | **12.70** |  |  | **22.16** |
| *B. rapa* OR | | 33.81 | ± 1.07 | 47.13 | ± 1.04 | 13.31 | 51.56 | ± 1.05 | 17.75 |
| *B. rapa* CA | | 57.20 | ± 2.15 | 67.90 | ± 2.99 | 10.70 | 70.33 | ± 2.12 | 13.13 |
|  | *Average B. rapa* |  |  |  |  | **12.01** |  |  | **15.44** |
| *B. juncea* | | 30.12 | ± 0.24 | 48.07 | ± 1.67 | 17.95 | 52.43 | ± 2.34 | 22.31 |
| *B. nigra* | | 45.79 | ± 3.59 | 67.42 | ± 4.42 | 21.63 | 75.25 | ± 5.99 | 29.46 |
